# Association between being metabolically healthy/unhealthy and metabolic syndrome in Iranian adults

**DOI:** 10.1371/journal.pone.0262246

**Published:** 2022-01-06

**Authors:** Ozra Tabatabaei-Malazy, Sahar Saeedi Moghaddam, Masoud Masinaei, Nazila Rezaei, Sahar Mohammadi Fateh, Arezou Dilmaghani-Marand, Elham Abdolhamidi, Farideh Razi, Patricia Khashayar, Alireza Mahdavihezaveh, Siamak Mirab Samiee, Bagher Larijani, Farshad Farzadfar

**Affiliations:** 1 Non-Communicable Diseases Research Center, Endocrinology and Metabolism Population Sciences Institute, Tehran University of Medical Sciences, Tehran, Iran; 2 Endocrinology and Metabolism Research Center, Endocrinology and Metabolism Clinical Sciences Institute, Tehran University of Medical Sciences, Tehran, Iran; 3 Departments of Epidemiology and Biostatistics, Tehran University of Medical Sciences, Tehran, Iran; 4 Diabetes Research Center, Endocrinology and Metabolism Clinical Sciences Institute, Tehran University of Medical Sciences, Tehran, Iran; 5 Osteoporosis Research Center, Endocrinology and Metabolism Clinical Sciences Institute, Tehran University of Medical Sciences, Tehran, Iran; 6 Center for Microsystems Technology, Imec and Ghent, University Ghent Belgium, Ghent, Belgium; 7 Deputy of Health, Ministry of Health and Medical Education, Tehran, Iran; 8 Reference Health Laboratory, Ministry of Health and Medical Education, Tehran, Iran; Tabriz University of Medical Sciences, ISLAMIC REPUBLIC OF IRAN

## Abstract

**Introduction:**

The prevalence of metabolically healthy obesity (MHO) varies based on different criteria. We assessed the prevalence of MHO and metabolic unhealthiness based on body mass index (BMI) and their association with metabolic syndrome (MetS) in a nation-wide study.

**Methods:**

Data were taken from the STEPs 2016 study, from 18,459 Iranians aged ≥25 years. Demographic, metabolic, and anthropometric data were collected. Subjects were stratified by BMI, metabolic unhealthiness, and having MetS. The latter was defined based on National Cholesterol Education Program Adult Treatment Panel III 2004 (NCEP ATP III), was then assessed.

**Results:**

The prevalence of MHO and metabolic unhealthiness in obese subjects was 7.5% (about 3.6 million) and 18.3% (about 8.9 million), respectively. Most of the metabolic unhealthy individuals were female (53.5%) or urban residents (72.9%). Low physical activity was significantly and positively associated (Odds Ratio: 1.18, 95% CI: 1.04–1.35) with metabolic unhealthiness, while being a rural residence (0.83, 0.74–0.93), and having higher education (0.47, 0.39–0.58) significantly but negatively affected it. Dyslipidemia was the most frequent MetS component with a prevalence rate of 46.6% (42.1–51.1), 62.2% (60.8–63.6), 76.3% (75.1–77.5), and 83.4% (82.1–84.6) among underweight, normal weight, overweight and obese phenotypes, respectively.

**Conclusion:**

BMI aside, an additional set of criteria such as metabolic markers should be taken into account to identify normal weight but metabolically unhealthy individuals. Given the highest prevalence of dyslipidemia among obese subjects, further interventions are required to raise public awareness, promote healthy lifestyles and establish lipid clinics.

## Introduction

Being overweight and/or obese accelerates the incidence and mortality rate of many non-communicable diseases (NCDs) such as type 2 diabetes mellitus (T2DM), and cardiovascular diseases (CVD) [1–3]. Some studies have shown a variation in this regard by taking into account body size phenotypes and metabolic profiles. In other words, some obese individuals, whom despite their high body mass index (BMI) do not have any cardiometabolic risk factors, are known as metabolically healthy obese (MHO) [4, 5]. This is while some normal weight individuals have CVD risk factors; they, therefore, are considered to be metabolically unhealthy normal weight (MUHNW) [6].

The prevalence rate of such phenotypes is different based on the definition criteria. Globally, MHO individuals represent 10–45% of the adult obese population based on diagnostic criteria, with higher prevalence among younger obese individuals in general and women [7]. Among US adults aged ≥20 years, about 10% of the US population (31.7% of all obese individuals) have been classified as MHO based on having ≤2 metabolic abnormalities, as defined by the National Health and Nutrition Examination Survey (NHANES) 1999–2004 [8]. However, Wildman et al used having 0 or 1 metabolic abnormalities to identify MHO individuals in NHANES 2005–2012, and reported that about 9.0% of the population (26.42% of all obese individuals) was MHO [8, 9].

A high number of Iranians were reported to be overweight and obese based on STEPs 2016, suggesting that prevention and control of obesity in this population through serious interventional strategies is required [10]. As a result, accurate illustration of the metabolic status of the overweight and obese people in this population is needed. To our knowledge, no study has reported the frequency of MHO in a population-based study, representative of the Iranian population. Hence, the aim of the present study is to assess the prevalence rate of metabolically healthy/unhealthy individuals based on BMI categories as well as their association with metabolic syndrome (MetS) in a nation-wide study on the Iranian adults.

## Materials & methods

### Study design and participants

Data were taken from the STEPs 2016 study, whose protocol has been published elsewhere [11]. In brief, this cross-sectional study was conducted on the Iranian adults in 2016. Required data was collected using questionnaires, and the results of anthropometric measurements and blood serum evaluations. Study participants gave their written informed consent. The study was ethically approved by Ethical Committee of National Institute for Medical Research Development (ID: IR.NIMAD.REC.1397.513).

### Study variables

Weight, height, BMI, waist circumference (WC), waist-to-hip-ratio (WHR), systolic (SBP) and diastolic blood pressure (DBP) were measured in each subject by trained staff under standard protocols previously described in the STEPs manual [12, 13]. Weight was measured by Innofit, JY-218A personal scale, SN: 14010936 (China). BP was measured by Beurer sphingometer, Type: BM 20, Art_Nr: 652.11 (Germany).

The participants’ age, educational status (Illiterate, 1–6 years, 7–12 years, 13 years and more), job (employed, unemployed, homemaker), marital status and smoking habits were self-reported. Physical activity was measured by the Global Physical Activity Questionnaire (GPAQ) and reported as Metabolic Equivalent of Task (MET) [14].

Venous blood samples were taken after 12–14 hours of overnight fasting to measure fasting blood sugar (FBS), total cholesterol (TChol), high density lipoprotein cholesterol (HDL-C), low density lipoprotein cholesterol (LDL-C), and triglycerides (TGs). In patients with TGs <400 mg/dl, LDL-C was estimated by the Friedewald formula (TChol minus HDL-C minus TGs/5 in mg/dl), whereas it was measured directly in the others [15]. HDL-C was measured using the homogeneous enzymatic cardiometric test. All the collected samples were assessed using kits with definite batch numbers in the Non-Communicable Diseases Research Center (NCDRC) laboratory.

The collected samples were stored under standard conditions (at temperatures lower than 4°C) in vaccine transfer boxes and were transferred to the central processing/archiving laboratory of study in the NCDRC of Endocrinology and Metabolism Population Sciences Institute of Tehran University of Medical Sciences in the shortest possible time (less than 18 hours). During transfer, a digital thermometer recorded the temperature in each cold box. FBS, TChol, and TGs levels were tested following a standard enzymatic method using an auto-analyzer (Cobas C311, Hitachi, Japan).

### Definitions

BMI was subdivided into 4 categories <18.5, 18.5–24.99, 25.0–29.99, and ≥30 kg/m^2^, known as underweight, normal weight, overweight, and obese, respectively [16]. Low physical activity was defined according to WHO’s recommendation of less than 600 METs per week [17]. MetS was defined based on the National Cholesterol Education Program Adult Treatment Panel III 2004 (NCEP ATP III) criteria [18]. Being metabolically unhealthy was referred to individuals with more than one of the following criteria: FBS ≥100 mg/dl (or diagnosed diabetes), TGs ≥150 mg/dl, HDL-C <40 mg/dl in men and <50 mg/dl in women, SBP ≥130 mmHg and DBP ≥85 mmHg. By stratifying metabolic phenotypes and BMI categories, we generated eight body size phenotypes: (i) underweight with normal metabolic profiles, called metabolically healthy underweight phenotype (MHUW), (ii): underweight with abnormal metabolic profiles, called metabolically unhealthy underweight phenotype (MUHUW), (iii): normal weight with normal metabolic profiles, called metabolically healthy normal weight phenotype (MHNW), (iiii): normal weight with abnormal metabolic profiles, called metabolically unhealthy normal weight phenotype (MUHNW), (v): overweight with normal metabolic profiles, called metabolically healthy overweight phenotype (MHOW), (vi): overweight with abnormal metabolic profiles, called metabolically unhealthy overweight phenotype (MUHOW), (vii): obese with normal metabolic profiles, called metabolically healthy obese phenotype (MHO), and (viii): obese with abnormal metabolic profiles, called metabolically unhealthy obese phenotype (MUHO).

### Statistical analysis

Upon conducting complex survey analysis, the demographic, lifestyle and biochemical characteristics of the participants within each body size phenotype were presented as overall and point estimates along with their 95% confidence interval (95% CI). We evaluated the statistical difference between the prevalence of being metabolically unhealthy and healthy normal-weight using other BMI categories and logistic or linear regressions. Logistic regression was used to calculate unadjusted and adjusted Odds Ratio (OR) for having ≥2 cardiometabolic abnormalities among normal weight individuals or having <2 cardiometabolic abnormalities among overweight and obese subjects. Sex, resident area, age, educated years, low physical activity, current daily cigarette smoking and WC were used for reporting the adjusted ORs. Sensitivity analysis was done to calculate the prevalence of being metabolically unhealthy/healthy based on abdominal obesity and to explore the association between the metabolically unhealthy phenotypes among the Iranian population. The Package survey in R version 3.4.0 was used to estimate the weighted prevalence and plot the figures [19].

## Results

This study was conducted on 18,459 participants, including 8,594 male and 9,865 female aged ≥25 years (mean age: 47.6, 95% CI: 47.4–47.9). The demographic and metabolic characteristics of the participants based on their body size phenotypes are presented in Table 1. Most of the metabolically unhealthy subjects were older than 47.0 years, married, hypertensive, with some 7–12 years of educations, had low physical activity, greater WC, and suffered from biochemical abnormalities, Table 1.

**Table 1:** Comparison of the demographical and metabolic characteristics of the participants based on their body size phenotype.

Variable	Overall N = 18,459	Underweight		Normal weight		Overweight		Obese	
		Metabolically Unhealthy (MUHUW) N = 140	Metabolically Healthy (MHUW) N = 492	Metabolically Unhealthy (MUHNW) N = 2,118	Metabolically Healthy (MHNW) N = 3,964	Metabolically Unhealthy (MUHOW) N = 4,011	Metabolically Healthy (MHOW) N = 3,074	Metabolically Unhealthy (MUHO) N = 3,289	Metabolically Healthy (MHO) N = 1,371
**Female (%)**	54.1 (53.2–54.9)	44.4 (35.8–53.0)	43.6 (38.4–48.9)	44.2 (41.6–46.8)	45.6 (43.7–47.4)	46.1 (44.2–48.0)	58.6 (56.6–60.7) ^a^	68.7 (66.9–70.5) ^a^	74.5 (71.9–77.1) ^a^
**Mean Age (yrs.)**	47.6 (47.4–47.9)	53.2 (49.9–56.4)	43.9 (41.7–46.1)	51.5 (50.6–52.3)	42.8 (42.3–43.4)	51.3 (50.8–51.8)	43.5 (43.0–44.0)	51.0 (50.5–51.5)	46.1 (45.3–46.9) ^a^
**Educated years (%)**									
Illiterate	16.8 (16.3–17.4)	39.0 (30.6–47.4) ^a^	19.7 (15.9–23.5) ^b^	22.8 (20.8–24.8)	13.5 (12.4–14.7)	18.4 (17.1–19.7) ^a^	10.4 (9.3–11.6) ^a^	20.7 (19.2–22.3)	14.0 (12.0–16.0)
1–6 years	28.7 (27.9–29.4)	23.5 (16.3–30.7)	27.5 (23.1–31.9)	26.9 (24.7–29.2)	24.9 (23.3–26.5)	29.2 (27.5–30.9)	25.4 (23.6–27.1)	34.4 (32.5–36.3) ^a^	34.4 (31.5–37.4) ^a^
7–12 years	37.8 (37.0–38.7)	18.0 (11.3–24.7) ^a^	38.5 (33.1–43.9)	34.1 (31.5–36.6)	40.0 (38.2–41.8)	36.5 (34.7–38.4)	44.2 (42.1–46.4) ^b^	34.0 (32.0–35.9)	38.1 (35.1–41.2)
13 years and more	16.6 (16.0–17.3)	19.5 (11.9–27.1)	14.3 (10.6–18.0) ^b^	16.2 (14.2–18.2)	21.5 (19.9–23.2)	15.9 (14.4–17.3)	19.9 (18.3–21.6)	10.9 (9.7–12.1) ^a^	13.5 (11.3–15.6) ^a^
**Job (%)**									
Employed	38.1 (37.3–38.9)	34.5 (26.2–42.8)	40.9 (35.9–45.9) ^b^	40.2 (37.7–42.8)	48.3 (46.4–50.1)	40.7 (38.9–42.5)	38.7 (36.7–40.8) ^a^	27.1 (25.4–28.8) ^a^	23.9 (21.4–26.4) ^a^
Home maker	46.7 (45.8–47.5)	35.6 (27.4–43.8)	33.7 (28.6–38.8)	38.4 (35.9–41.0)	36.1 (34.4–37.9)	41.1 (39.2–42.9)	49.6 (47.5–51.8) ^a^	62.5 (60.6–64.4) ^a^	64.6 (61.5–67.6) ^a^
Unemployed	15.3 (14.6–15.9)	29.9 (21.6–38.3) ^c^	25.4 (20.5–30.4) ^a^	21.3 (19.2–23.4)	15.6 (14.2–17.0)	18.2 (16.8–19.7) ^c^	11.6 (10.4–12.9) ^a^	10.4 (9.3–11.6) ^a^	11.5 (9.0–14.1) ^c^
**Marital status (%)**									
Divorced	2.0 (1.7–2.3)	3.3 (0.4–6.3)	2.4 (0.9–4.0)	1.6 (1.0–2.2)	2.2 (1.5–2.9)	1.3 (0.9–1.7)	2.7 (1.7–3.7)	2.2 (1.6–2.8)	2.1 (1.2–3.1)
Married	83.4 (82.7–84)	65.7 (57.5–74.0) ^a^	71.5 (67.0–76.0) ^a^	81.2 (79.2–83.1)	79.8 (78.2–81.3)	86.6 (85.3–87.8) ^a^	85.4 (83.7–87) ^a^	83.5 (81.9–85.2)	87.8 (85.8–89.8) ^a^
Never married	7.4 (7.0–7.9)	12.1 (6.1–18.1)	21.0 (17.0–25.1) ^a^	8.0 (6.7–9.3)	14.1 (12.8–15.4)	4.5 (3.8–5.2) ^a^	7.3 (6.3–8.3) ^a^	2.7 (2.0–3.3) ^a^	3.9 (2.9–5.0) ^a^
Widowed	7.1 (6.7–7.6)	18.8 (12.3–25.3) ^a^	5.1 (3.1–7.0)	9.2 (7.7–10.7)	3.9 (3.2–4.7)	7.6 (6.6–8.6)	4.6 (3.7–5.6)	11.5 (10.1–13.0) ^c^	6.2 (4.6–7.7) ^b^
**Having basic insurance (%)**	93.6 (93.2–94.1)	88.9 (82.7–95.1)	93.1 (90.6–95.6)	93.7 (92.4–95.1)	92.3 (91.2–93.5)	94.0 (93.2–94.9)	94.0 (92.8–95.2) ^c^	93.7 (92.6–94.7)	95.4 (94.3–96.6) ^a^
**Having complementary insurance (%)**	22.2 (21.5–23.0)	10.8 (5.1–16.5) ^c^	10.4 (6.3–14.6) ^c^	20.6 (18.5–22.7)	16.0 (14.5–17.5)	26.3 (24.6–28.0) ^a^	21.7 (19.9–23.5) ^a^	26.6 (24.7–28.4) ^a^	25.2 (22.3–28.2) ^a^
**Wealth index quintile (%)**									
First quintile (Poorest)	19.7 (19.1–20.3)	62.7 (54.0–71.3) ^a^	44.4 (39.2–49.6) ^a^	24.6 (22.6–26.7)	26.4 (24.9–27.9)	16.2 (14.9–17.5) ^a^	17.6 (16.0–19.2) ^a^	12.7 (11.4–13.9) ^a^	13.6 (11.8–15.4) ^a^
Second quintile (Poorer)	19.9 (19.3–20.6)	16.2 (9.7–22.7)	19.8 (16.0–23.6)	21.9 (19.8–23.9)	20.9 (19.4–22.4)	19.3 (17.9–20.7) ^c^	17.2 (15.6–18.7) ^b^	20.9 (19.3–22.6)	20.4 (17.7–23.1)
Third quintile (Middle)	20.5 (19.8–21.2)	6.7 (2.7–10.7) ^a^	16.3 (11.8–20.8)	20.4 (18.4–22.4)	18.9 (17.3–20.4)	19.8 (18.4–21.2)	20.1 (18.4–21.8)	24.1 (22.3–25.9) ^b^	22.0 (19.5–24.5) ^c^
Fourth quintile (Richer)	19.8 (19.1–20.5)	5.9 (1.7–10.0) ^b^	12.5 (9.2–15.8) ^b^	17.0 (15.1–18.9)	18.5 (17.0–20.0)	21.4 (19.9–23.0) ^b^	20.0 (18.4–21.7)	21.7 (20.0–23.3) ^a^	21.1 (18.6–23.6)
Fifth quintile (Richest)	20.1 (19.4–20.8)	8.6 (2.4–14.7)	7.1 (4.1–10.0) ^a^	16.1 (13.8–18.4)	15.4 (14.0–16.7)	23.3 (21.5–25.0) ^a^	25.0 (23.1–27.0) ^a^	20.6 (19.0–22.3) ^b^	22.9 (20.2–25.6) ^a^
**Low physical activity (<600 METs) (%)**	56.5 (55.5–57.4)	58.1 (49.1–67.2)	50.2 (44.4–56.1)	57.1 (54.4–59.8)	53.4 (51.3–55.4)	56.1 (54.2–58.1)	53.6 (51.3–55.8)	62.0 (60.0–64.0) ^b^	58.5 (55.3–61.7) ^b^
**Current daily cigarette smoker (%)**	9.8 (9.3–10.3)	18.6 (11.8–25.5)	18.0 (14.3–21.6) ^c^	14.8 (13.1–16.6)	13.5 (12.2–14.7)	10.1 (9.0–11.1) ^a^	7.2 (6.2–8.2) ^a^	5.5 (4.6–6.4) ^a^	4.3 (3.0–5.6) ^a^
**Waist circumference (cm)**	92.3 (92.1–92.5)	73.8 (72.1–75.6) ^a^	70.4 (69.4–71.3) ^a^	85.3 (84.7–85.9)	81.1 (80.7–81.5)	95.1 (94.7–95.5) ^a^	91.7 (91.3–92.1) ^a^	105.9 (105.5–106.3) ^a^	102.5 (101.8–103.2) ^a^
**Being abdominal obese (WC ≥102 cm (men); ≥ 88 cm (women)) (%)**	45.3 (44.5–46.2)	3.2 (0.3–6.2) ^a^	0.4 (0.0–0.9) ^a^	17.5 (15.5–19.5)	8.6 (7.5–9.7)	49.7 (47.9–51.6) ^a^	42.8 (40.7–44.9) ^a^	91.4 (90.3–92.5) ^a^	87.7 (85.6–89.7) ^a^
WHR (*100)	90.6 (90.4–90.8)	86.3 (84.6–88.0) ^a^	82.8 (81.9–83.6) ^a^	90.2 (89.7–90.7)	86.8 (86.4–87.2)	93.1 (92.7–93.5) ^a^	89.6 (89.3–90.0) ^a^	93.9 (93.5–94.2) ^a^	91.3 (90.7–91.8) ^a^
**Weight (kg)**	72.2 (72.0–72.5)	46.3 (45.1–47.5) ^a^	47.1 (46.5–47.7) ^a^	62.6 (62.1–63.1)	61.0 (60.7–61.3)	74.5 (74.2–74.9) ^a^	72.9 (72.5–73.2) ^a^	86.9 (86.4–87.4) ^a^	84.6 (83.9–85.3) ^a^
**BMI (Kg/m** ^**2**^ **)**	27.1 (27.0–27.2)	17.2 (17.0–17.4) ^a^	17.2 (17.1–17.3) ^a^	22.9 (22.8–23.0)	22.3 (22.2–22.4)	27.5 (27.5–27.6) ^a^	27.2 (27.2–27.3) ^a^	33.8 (33.7–34.0) ^a^	33.1 (32.9–33.3) ^a^
**SBP (mmHg)**	127.2 (126.9–127.5)	132.4 (129.1–135.7)	114.2 (111.7–116.7) ^c^	132.9 (131.8–134.0)	116.9 (116.4–117.4)	134.7 (133.9–135.4)^b^	119.2 (118.7–119.8) ^a^	137.3 (136.5–138.1) ^a^	121.3 (120.4–122.2) ^a^
**DBP (mmHg)**	78.0 (77.8–78.2)	79.3 (77.6–81.0)	70.0 (68.5–71.5) ^a^	80.3 (79.7–80.8)	72.8 (72.5–73.2)	82.0 (81.5–82.4) ^a^	74.5 (74.1–74.8) ^a^	83.1 (82.7–83.6) ^a^	75.3 (74.7–75.9) ^a^
**Being HTN (SBP ≥ 130 mmHg or DBP ≥ 85 mmHg) (%)**	41.9 (41.0–42.7)	63.5 (54.9–72.1)	12.2 (8.0–16.4)	63.0 (60.6–65.4)	13.6 (12.4–14.8)	65.3 (63.5–67.1)	14.8 (13.3–16.4)	69.9 (68.1–71.8) ^a^	17.9 (15.6–20.2) ^b^
**FBS (mg/dl)**	99.6 (98.9–100.3)	100.2 (94.0–106.5)	86.2 (83.8–88.5)	106.8 (104.4–109.3)	88.2 (87.1–89.3)	109.0 (107.3–110.6)	89.4 (88.8–90.0)	111.8 (109.9–113.7) ^b^	89.6 (88.7–90.5)
**Being diabetics (FBS ≥ 100 mg/dl includes diabetes) (%)**	29.2 (28.4–30.0)	42.5 (33.9–51.1)	6.8 (4.3–9.2)	46.6 (44.0–49.2)	6.0 (5.0–7.1)	48.7 (46.8–50.5)	6.0 (5.1–6.9)	53.9 (51.9–55.9) ^a^	6.7 (5.2–8.2)
**TChol (mg/dl)**	164.5 (163.9–165.2)	156.3 (149.1–163.4) ^c^	144.2 (141.0–147.5) ^a^	165.6 (163.7–167.5)	154.0 (152.8–155.2)	170.9 (169.6–172.2) ^a^	160.8 (159.5–162.1) ^a^	174.3 (172.8–175.7) ^a^	165.4 (163.5–167.3) ^a^
**TGs (mg/dl)**	129.2 (127.8–130.6)	115.6 (101.2–130.0) ^a^	69.9 (67.2–72.6) ^a^	149.4 (145.3–153.5)	83.0 (81.9–84.2)	169.3 (165.4–173.3) ^a^	93.5 (91.9–95.0) ^a^	173.7 (170.1–177.3) ^a^	97.7 (95.5–99.9) ^a^
**Being hypertriglyceridemia (TGs ≥ 150 mg/dl) (%)**	27.7 (26.9–28.5)	22.3 (14.6–30.0) ^a^	0.9 (0.0–2.2)	42.5 (39.9–45.0)	1.6 (1.0–2.1)	52.1 (50.3–54.0) ^a^	3.3 (2.5–4.2) ^b^	54.6 (52.5–56.6) ^a^	2.9 (1.9–4.0) ^c^
**HDL-C (mg/dl)**	41.2 (41.0–41.4)	38.5 (36.7–40.2)	48.7 (47.6–49.7) ^a^	36.9 (36.4–37.3)	46.5 (46.0–46.9)	36.3 (36.0–36.7) ^c^	45.2 (44.7–45.7) ^a^	37.1 (36.7–37.5)	46.2 (45.5–46.9)
**Low HDL-C < 40 mg/dl (men)/; < 50 mg/dl (women) (%)**	69.3 (68.5–70.1)	89.7 (84.7–94.7)	34.7 (29.9–39.4) ^b^	89.1 (87.6–90.7)	43.6 (41.7–45.4)	88.6 (87.3–89.9)	50.9 (48.8–53.1) ^a^	90.3 (89.1–91.4)	52.4 (49.3–55.5) ^a^
**LDL-C (mg/dl)**	97.7 (97.2–98.2)	95.5 (89.1–101.9)	81.6 (78.8–84.4) ^a^	98.9 (97.3–100.5)	90.9 (89.9–92.0)	101 (99.9–102.2) ^c^	96.9 (95.8–98.1) ^a^	102.8 (101.5–104.1) ^a^	99.8 (98.1–101.5) ^a^

**WC**: Waist Circumferences; **WHR**: Waist Hip Ratio; **BMI**: Body Mass Index; **SBP**: Systolic Blood Pressure; **DBP**: Diastolic Blood Pressure; **HTN**: Hypertension; **FBS**: Fasting Blood Sugar; **TChol**: Total Cholesterol; **TGs**: Triglycerides; **HDL-C**: High Density Lipoprotein-Cholesterol; **LDL-C**: Low Density Lipoprotein-Cholesterol.

- Data presented as point estimation (prevalence or mean) and its 95% CI.

- By survey logistic or linear regression models

a p < 0.001 versus normal weight within metabolic subgroup.

b p < 0.01 versus normal weight within metabolic subgroup.

c p < 0.05 versus normal weight within metabolic subgroup.

The sex pattern of metabolically unhealthy individuals shifted from women of the under-, normal- and overweight group to obese men. The prevalence of being metabolically unhealthy was significantly different between obese men and women only. Among all BMI groups, the mean age of metabolically unhealthy individuals was older than metabolically healthy ones. The educational pattern of metabolically unhealthy subjects shifted from illiteracy (16.8%) to having 7–12 years of schooling (37.8%). This pattern was regardless of BMI categories (ranging from 39.0%, 34.1%, 36.5%, 34.0% among underweight, normal weight, overweight, and obese illiterate subjects to 18.0%, 34.1%, 36.5% and 34.0%, among subjects with similar BMI groups but with 7–12 years of education, respectively). In addition, the highest prevalence of being metabolically healthy was seen in underweight, normal weight, overweight and obese subjects with 7–12 years of education; 38.5%, 40.0%, 44.2%, and 38.1%, respectively, Table 1.

Job pattern differed among different BMI categories; 35.6%, 41.1%, and 62.5% of metabolically unhealthy individuals were homemaker, with an increasing rate seen within the underweight, overweight and obese group, respectively. The highest number of metabolic unhealthiness in the employed subjects was 40.7%, and was observed among those with the overweight phenotype. Being metabolically healthy, on the other hand, was more prevalent among employed underweight and normal weight individuals, 40.9%, and 48.3%, respectively. Corresponding figures for overweight or obese phenotypes were 49.6% and 64.6%, respectively, and more frequently reported seen among the homemakers, Table 1.

The prevalence of abnormalities in the anthropometric and biochemical parameters was significantly higher with increasing BMI. The prevalence of MetS in the overall population, MUHUW, MUHNW, MUHOW, and MUHO groups were estimated at 38.3% (37.5–39.2), 19.3% (12.6–26.0), 44.5% (41.9–47.1), 70.1% (68.4–71.9), and 95.7% (94.9–96.5), respectively.

The prevalence of metabolically unhealthy phenotypes in the studied population (aged ≥25) was 52.6% (51.8–53.5; 25.7 million persons). Most of them were female (53.5%) or urban residents (72.9%). Based on the BMI categories, 0.6% of underweight (0.3 million people), 11.5% of normal weight (5.6 million people), 22.2% of overweight (10.9 million people), and 18.3% of obese individuals (8.9 million people) were metabolically unhealthy, Table 2. The percentage of being MHUW, MHNW, MHOW and MHO in the studied population were estimated at 2.5% (1.2 million people), 20.5% (10.0 million people), 16.8% (8.2 million people), and 7.5% (3.6 million people), respectively.

**Table 2:** Comparison of burden and prevalence of metabolic unhealthiness/healthiness among Iranian adults of different body size phenotype (for all ages) based on their living area (rural vs urban) and gender.

Participants	Type	Underweight		Normal weight		Overweight		Obese	
		Metabolically Unhealthy (MUHUW)	Metabolically Healthy (MHUW)	Metabolically Unhealthy (MUHNW)	Metabolically Healthy (MHNW)	Metabolically Unhealthy (MUHOW)	Metabolically Healthy (MHOW)	Metabolically Unhealthy (MUHO)	Metabolically Healthy (MHO)
**Total**	**Prevalence (%)**	0.6 (0.5–0.7)	2.5 (2.2–2.8)	11.5 (11.0–12.1)	20.5 (19.9–21.2)	22.2 (21.5–22.9)	16.8 (16.2–17.5)	18.3 (17.6–19.0)	7.5 (7.0–7.9)
	**Burden** *	308.0 (254.5–361.5)	1,220.8 (1,094.4–1,347.3)	5,631.7 (5,357.3–5,906.2)	10,041.7 (9,709.1–10,374.4)	10,861.3 (10,505.3–11,217.3)	8,228.9 (7,911.3–8,546.5)	8,943.3 (8,617.7–9,268.9)	3,645.9 (3,426.6–3,865.1)
**Rural**	**Prevalence (%)**	1.2 (1.0–1.5)	4.4 (3.9–5.0)	12.8 (11.9–13.7)	25.6 (24.4–26.7)	18.7 (17.6–19.7)	15.7 (14.7–16.7)	15.2 (14.2–16.2)	6.4 (5.8–7.1)
	**Burden** *	155.6 (136.6–177.2)	558.2 (523.6–597.3)	1,518.4 (1,434.0–1,602.0)	3,111.5 (3,014.9–3,208.5)	2,170.7 (2,056.3–2,282.8)	1,850.7 (1,748.7–1,950.7)	1,779.1 (1,671.5–1,884.2)	756.1 (687.9–822.1)
**Urban**	**Prevalence (%)**	0.4 (0.3–0.5)	1.7 (1.4–2.0)	11.0 (10.3–11.7)	18.4 (17.6–19.3)	23.7 (22.8–24.7)	17.3 (16.5–18.2)	19.6 (18.8–20.5)	7.9 (7.3–8.5)
	**Burden** *	152.4 (117.9–184.2)	662.7 (570.8–750.0)	4,113.3 (3,923.3–4,304.2)	6,930.2 (6,694.2–7,165.8)	8,690.6 (8,449.0–8,934.4)	6,378.3 (6,162.6–6,595.8)	7,164.2 (6,946.2–7,384.8)	2,889.8 (2,738.8–3,043.0)
**Female**	**Prevalence (%)**	0.5 (0.4–0.6)	2.0 (1.7–2.3)	9.4 (8.7–10.1)	17.3 (16.4–18.2)	18.9 (18.0–19.9)	18.3 (17.3–19.2)	23.2 (22.2–24.3)	10.3 (9.6–11.0)
	**Burden** *	123.4 (100.6–146.0)	479.6 (422.4–536.2)	2,246.5 (2,122.6–2,369.8)	4,138.0 (3,980.7–4,294.9)	4,529.3 (4,361.6–4,696.6)	4,465.4 (4,307.7–4,623.5)	5,788.2 (5,619.9–5,957.7)	2,587.2 (2,463.1–2,712.7)
**Male**	**Prevalence (%)**	0.8 (0.6–0.9)	3.1 (2.6–3.5)	14.0 (13.1–14.9)	24.3 (23.3–25.4)	26.1 (25.0–27.2)	15.2 (14.3–16.0)	12.5 (11.7–13.3)	4.1 (3.7–4.6)
	**Burden** *	184.6 (154.0–215.5)	741.2 (672.0–811.0)	3,385.2 (3,234.7–3,536.4)	5,903.7 (5,728.4–6,079.5)	6,332.0 (6,143.7–6,520.7)	3,763.6 (3,603.7–3,923.0)	3,155.1 (2,997.8–3,311.2)	1,058.7 (963.6–1,152.4)

- Data presented as point estimation and its 95% CI.

* Rounded to the nearest thousand.

- All differences for rural versus urban area by each group were statistically significant (p < 0.05).

- All differences for female versus male by each group were statistically significant (p < 0.05).

**Fig 1:**
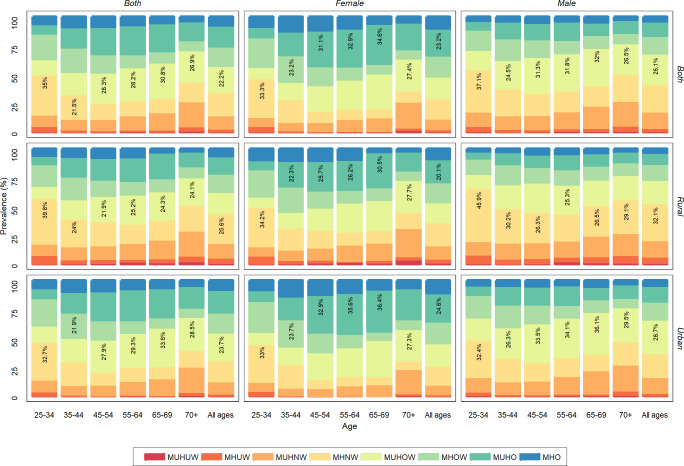
National prevalence of metabolic phenotypes for each BMI category by sex, area of residence, and age groups. MUHUW: metabolically unhealthy underweight, MHUW: metabolically healthy underweight, MUHNW: metabolically unhealthy normal weight, MHNW: metabolically healthy normal weight, MUHOW: metabolically unhealthy overweight, MHOW: metabolically healthy overweight, MUHO: metabolically unhealthy obese, MHO: metabolically healthy obese.

Based on national estimates by age group and sex (Fig 1), most Iranian females had the MUHO (34.6%), and this phenotype was more prevalent in the 65-69-year age group. As for the males, however, the MHNW (37.1%) was more frequent, and the phenotype was mainly reported in the 25-34-year age group. National estimates by area indicate that the majority of people in the urban and rural areas had the MUHOW or MHNW phenotypes, respectively, Fig 1.

**Fig 2:**
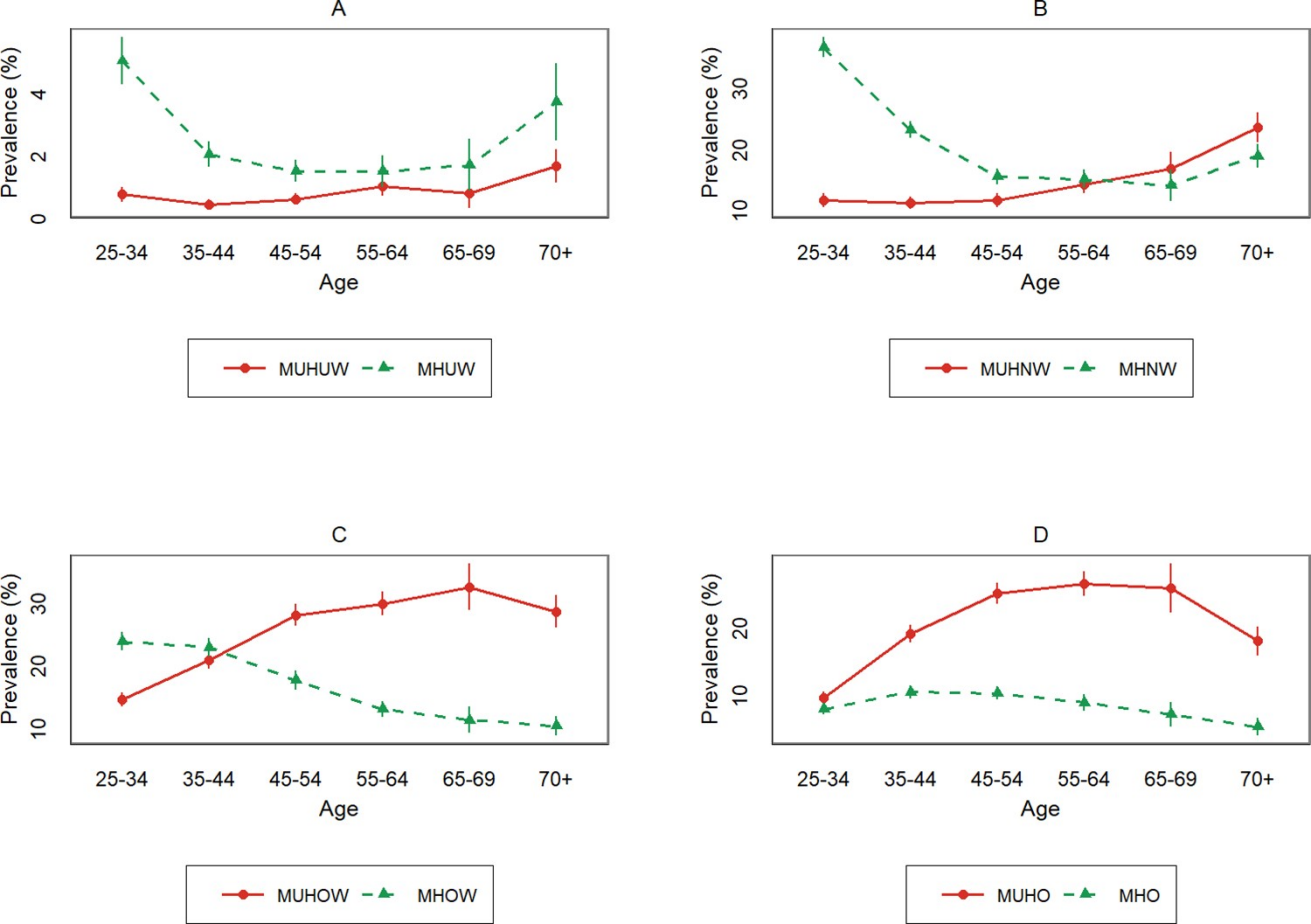
The prevalence of metabolically unhealthy phenotypes compared with the metabolically healthy phenotypes by age groups and body size. MUHUW: metabolically unhealthy underweight, MHUW: metabolically healthy underweight, MUHNW: metabolically unhealthy normal weight, MHNW: metabolically healthy normal weight, MUHOW: metabolically unhealthy overweight, MHOW: metabolically healthy overweight, MUHO: metabolically unhealthy obese, MHO: metabolically healthy obese.

The prevalence of metabolically unhealthy phenotypes was higher than that of metabolically healthy phenotypes in the 65–69 age groups within normal weight individuals. As for the subjects with overweight or obese phenotypes, this was observed in the 45–54 and 25–34 age groups, respectively, Fig 2. The prevalence of the metabolically unhealthy phenotype increased with BMI. The prevalence of the metabolically unhealthy phenotype was lower than that of the metabolically healthy phenotype in all age groups among underweight subjects.

**Fig 3:**
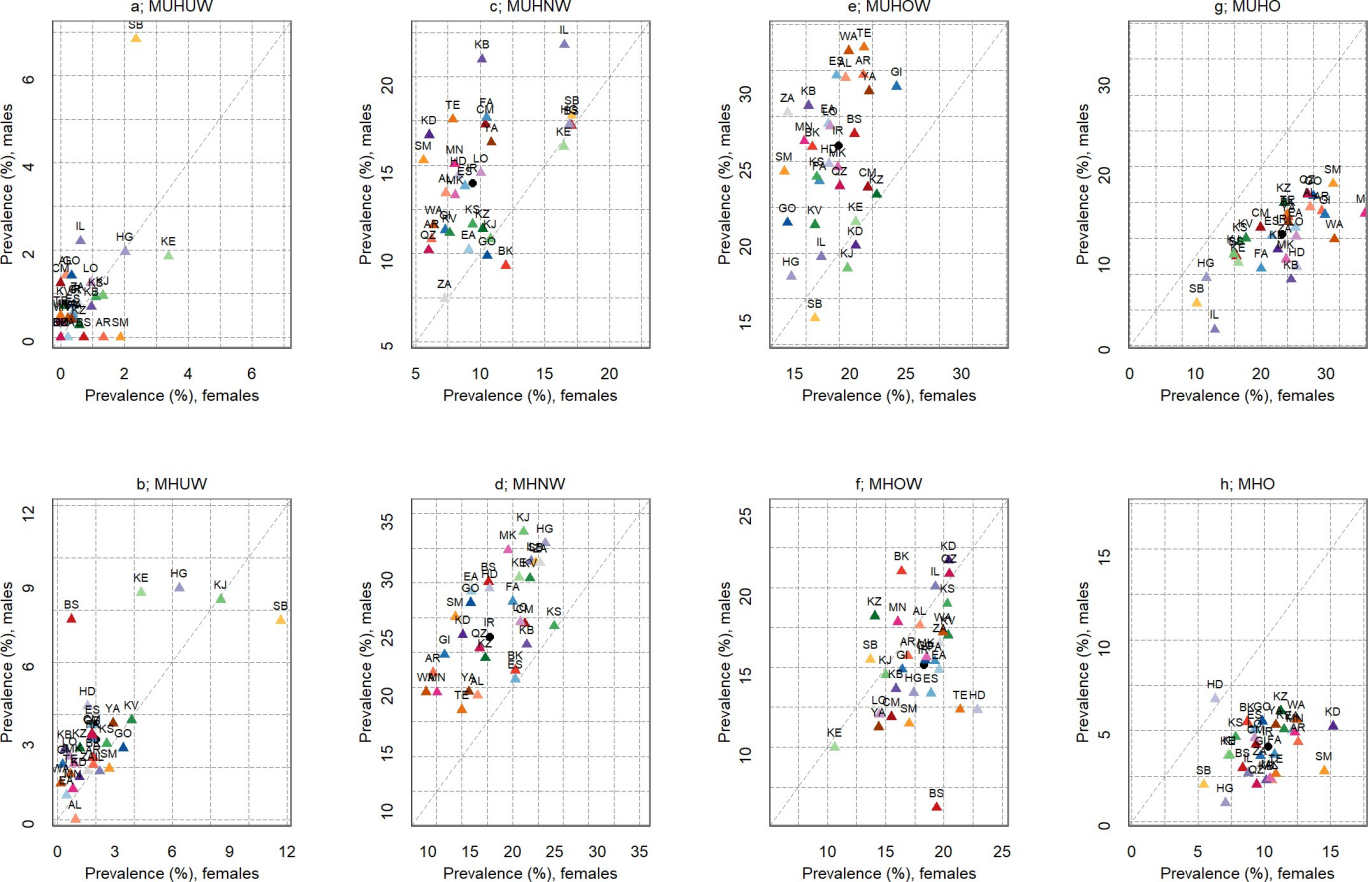
Sub-national prevalence of metabolic phenotypes for each BMI category by sex. (a) MUHUW: metabolically unhealthy underweight, (b) MHUW: metabolically healthy underweight, (c) MUHNW: metabolically unhealthy normal weight, (d) MHNW: metabolically healthy normal weight, (e) MUHOW: metabolically unhealthy overweight, (f) MHOW: metabolically healthy overweight, (g) MUHO: metabolically unhealthy obese, (h) MHO: metabolically healthy obese.

**Fig 4:**
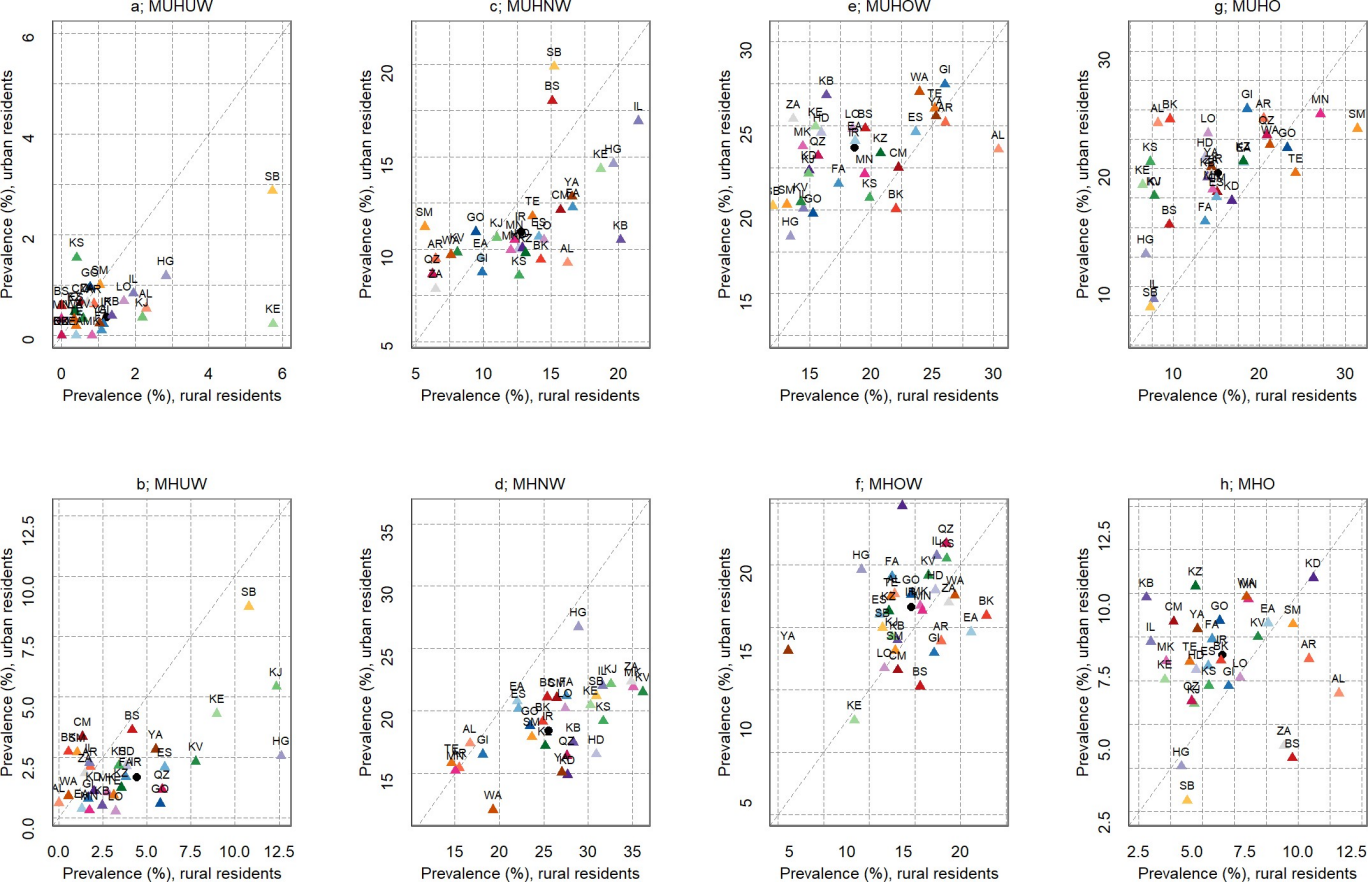
Sub-national prevalence of metabolic phenotypes for each BMI category by area of residence. (a) MUHUW: metabolically unhealthy underweight, (b) MHUW: metabolically healthy underweight, (c) MUHNW: metabolically unhealthy normal weight, (d) MHNW: metabolically healthy normal weight, (e) MUHOW: metabolically unhealthy overweight, (f) MHOW: metabolically healthy overweight, (g) MUHO: metabolically unhealthy obese, (h) MHO: metabolically healthy obese.

The prevalence of the metabolic phenotypes was different between female and male at sub-national level. The highest and lowest percentages of MHO among female subjects were reported in the western province, Kurdistan (15.2; 95% CI: 10.4–20.0) and the south-eastern province, Sistan-Baluchistan (5.5; 95% CI: 2.8–8.1), correspondingly. The highest and lowest percentage of MHO among male individuals was seen in the other western province, Hamadan (6.7; 95% CI: 3.1–10.4) and the south-western province, Hormozgan (1.0; 95% CI: 0.0–2.4), respectively. Overall, the most frequently observed metabolic phenotype among females was MUHO, which was mainly presented in the northern province, Mazandaran (35.9%). Among males, MHNW was most frequently observed (33.4%) in South Khorasan, Fig 3. Sub-national estimates by area indicated that the most frequent metabolic phenotype in the urban areas was MUHOW, and observed in the northern province of Guilan (27.5%), Fig 4. The most frequent metabolic phenotype observed in the rural areas was MUHNW, in the north-western province of Zanjan (34.9%), Fig 4.

Based on the number of MetS components stratified by BMI categories, most participants with underweight, normal weight, or overweight phenotypes had only one MetS component, whereas all obese subjects had atleast two MetS components, regardless of their sex or area of residence, (S1 Fig). The most frequent MetS component among our population, regardless of BMI, was dyslipidemia. The prevalence rate of dyslipidemia was 46.6% (42.1–51.1), 62.2% (60.8–63.6), 76.3% (75.1–77.5), and 83.4% (82.1–84.6) among individuals with underweight, normal weight, overweight and obese phenotypes, respectively.

Among normal weight individuals, the prevalence of metabolic unhealthiness was significantly higher among older ages than those aged 25-34-years (with a surging trend), particularly among individuals with low physical activity and large WC. It was significantly lower among rural residents and in more educated individuals. After adding WC to adjust the above-mentioned variables (sex, age, area of residence, years of education, low physical activity, current cigarette smoker), the association with residence in rural areas, age groups, and having a larger WC remained statistically significant, Table 3.

**Table 3:** Logistic regression model showing risk factors of metabolically unhealthy phenotype among normal weight individuals.

Variable	OR (95% CI) (unadjusted)	P-Value	OR (95% CI)^a^	P-Value^a^	OR (95% CI)^b^	P-Value^b^
**Sex**						
Female	Reference		Reference		Reference	
Male	1.05 (0.93–1.18)	0.467	1.05 (0.90–1.22)	0.553	0.93 (0.79–1.10)	0.399
**Resident area**						
Urban	Reference	0.002	Reference		Reference	
Rural	0.83 (0.74–0.93)		0.82 (0.71–0.94)	0.006	0.85 (0.73–0.98)	0.021
**Age**						
25–34 years	Reference		Reference		Reference	
35–44 years	1.57 (1.31–1.89)	<0.001	1.68 (1.37–2.06)	<0.001	1.54 (1.25–1.89)	<0.001
45–54 years	2.60 (2.14–3.16)	<0.001	2.86 (2.28–3.57)	<0.001	2.49 (1.99–3.12)	<0.001
55–64 years	3.45 (2.80–4.27)	<0.001	3.70 (2.87–4.75)	<0.001	3.02 (2.32–3.93)	<0.001
65–69 years	4.31 (3.17–5.86)	<0.001	4.68 (3.31–6.62)	<0.001	3.60 (2.50–5.17)	<0.001
70 years and more	4.28 (3.46–5.30)	<0.001	4.16 (3.17–5.45)	<0.001	3.40 (2.58–4.49)	<0.001
**Educated years**						
Illiterate	Reference		Reference		Reference	
1–6 years	0.64 (0.54–0.76)	<0.001	0.90 (0.73–1.11)	0.334	0.87 (0.71–1.08)	0.208
7–12 years	0.50 (0.43–0.59)	<0.001	0.90 (0.71–1.14)	0.378	0.87 (0.69–1.11)	0.262
13 years and more	0.47 (0.39–0.58)	<0.001	0.80 (0.62–1.04)	0.096	0.79 (0.60–1.03)	0.085
**Low physical activity (<600 METs)**						
No	Reference		Reference		Reference	
Yes	1.18 (1.04–1.35)	0.013	1.15 (1.00–1.32)	0.054	1.13 (0.98–1.30)	0.086
**Current daily cigarette smoking**						
No	Reference		Reference		Reference	
Yes	1.09 (0.92–1.29)	0.312	0.93 (0.75–1.15)	0.518	0.99 (0.80–1.22)	0.895
**Waist circumference**	1.05 (1.04–1.06)	<0.001			1.04 (1.03–1.05)	<0.001

^a^ Adjusted for sex, resident area, age, educated years, low physical activity, current daily cigarette smoking.

^b^ Adjusted for all above variables plus waist circumference.

Among overweight and obese individuals, older age groups compared to those aged between 25–34 years, males, those with low physical activity, current cigarette smokers, and those with a larger WC were less likely to be metabolically healthy. After adjusting for sex, age, area of residence, years of education, low physical activity, current cigarette smoker and WC, the negative association with sex, age, low physical activity, and having a larger WC remained statistically significant, Table 4.

**Table 4:** Logistic regression model showing risk factors of metabolically healthy phenotype among overweight and obese individuals.

Variable	OR (95% CI) (unadjusted)	P-Value	OR (95% CI)^a^	P-Value^a^	OR (95% CI)^b^	P-Value^b^
**Sex**						
Female	Reference		Reference		Reference	
Male	0.74 (0.68–0.81)	<0.001	0.66 (0.59–0.74)	<0.001	0.71 (0.64–0.80)	<0.001
**Resident area**						
Urban	Reference		Reference		Reference	
Rural	1.12 (1.02–1.23)	0.015	1.10 (0.99–1.22)	0.084	1.10 (0.98–1.22)	0.093
**Age**						
25–34 years	Reference		Reference		Reference	
35–44 years	0.60 (0.53–0.68)	<0.001	0.61 (0.53–0.70)	<0.001	0.65 (0.56–0.75)	<0.001
45–54 years	0.37 (0.32–0.42)	<0.001	0.38 (0.33–0.44)	<0.001	0.41 (0.35–0.48)	<0.001
55–64 years	0.26 (0.22–0.30)	<0.001	0.29 (0.24–0.34)	<0.001	0.33 (0.27–0.39)	<0.001
65–69 years	0.20 (0.16–0.26)	<0.001	0.23 (0.17–0.30)	<0.001	0.26 (0.20–0.34)	<0.001
70 years and more	0.21 (0.17–0.26)	<0.001	0.26 (0.20–0.33)	<0.001	0.29 (0.23–0.38)	<0.001
**Educated years**						
Illiterate	Reference		Reference		Reference	
1–6 years	1.51 (1.32–1.73)	<0.001	1.20 (1.02–1.40)	0.026	1.16 (0.99–1.36)	0.068
7–12 years	2.02 (1.77–2.31)	<0.001	1.36 (1.14–1.61)	0.001	1.25 (1.05–1.48)	0.012
13 years and more	2.23 (1.91–2.60)	<0.001	1.58 (1.30–1.92)	<0.001	1.45 (1.19–1.76)	<0.001
**Low physical activity (<600 METs)**						
No	Reference		Reference		Reference	
Yes	0.86 (0.78–0.94)	0.002	0.82 (0.75–0.91)	<0.001	0.85 (0.77–0.94)	0.001
**Current daily cigarette smoking**						
No	Reference		Reference		Reference	
Yes	0.77 (0.65–0.91)	0.002	0.93 (0.75–1.14)	0.467	0.93 (0.76–1.15)	0.512
**Waist circumference**	0.96 (0.96–0.97)	<0.001			0.97 (0.97–0.98)	<0.001

^a^ Adjusted for sex, resident area, age, educated years, low physical activity, current daily cigarette smoking.

^b^ Adjusted for all above variables plus waist circumference.

### Sensitivity analysis

When abdominal obesity (WC ≥102 cm in men and ≥88 cm in women) was used as a criterion, 22.9% of individuals without abdominal obesity were metabolically unhealthy (>1 cardiometabolic abnormality), whereas 15.5% of those with abdominal obesity fell into the metabolically healthy phenotype category. Among the overall population, the association between BMI categories and metabolic unhealthiness is outlined in Table 5. After adjusting for age and sex, the probability of being metabolically unhealthy was significantly and positively associated with low physical activity and BMI categories (Table 5, model 1). When ‘abdominal obesity’ was used instead of BMI categories in the logistic regression (model 1), the ‘low physical activity’ and ‘abdominal obesity’ remained statistically significant and positively associated with ≥2 cardiometabolic abnormalities. When ‘years of education’ was added to the equation (Table 5, model 2), the probability of being metabolically unhealthy was positively associated with low physical activity and BMI categories, and decreased with any increase in years of education. When abdominal obesity was added to the equation instead of BMI categories, residence in rural areas became negatively significant, and low physical activity, abdominal obesity and years of education remained significant.

**Table 5:** Sensitivity analysis to assess the association of different risk factors and risk of being metabolically unhealthy phenotype among the overall Iranian population.

Variable	Model 1				Model 2			
	OR (95% CI)	P-Value	OR (95% CI)	P-Value	OR (95% CI)	P-Value	OR (95% CI)	P-Value
**Sex**								
Female	Reference		Reference		Reference		Reference	
Male	1.17 (1.07–1.27)	0.001	1.45 (1.32–1.59)	<0.001	1.22 (1.11–1.34)	<0.001	1.50 (1.36–1.66)	<0.001
**Resident area**								
Urban	Reference		Reference		Reference		Reference	
Rural	0.99 (0.91–1.07)	0.753	0.93 (0.86–1.01)	0.085	0.93 (0.85–1.01)	0.085	0.88 (0.81–0.96)	0.003
**Age**								
25–34 years	Reference		Reference		Reference		Reference	
35–44 years	1.86 (1.65–2.11)	<0.001	1.95 (1.73–2.21)	<0.001	1.82 (1.60–2.06)	<0.001	1.91 (1.68–2.16)	<0.001
45–54 years	3.45 (3.04–3.92)	<0.001	3.56 (3.14–4.04)	<0.001	3.26 (2.86–3.71)	<0.001	3.37 (2.96–3.84)	<0.001
55–64 years	5.44 (4.75–6.24)	<0.001	5.23 (4.56–6.00)	<0.001	4.97 (4.29–5.75)	<0.001	4.83 (4.17–5.59)	<0.001
65–69 years	7.61 (6.24–9.28)	<0.001	6.87 (5.64–8.38)	<0.001	6.80 (5.52–8.37)	<0.001	6.24 (5.07–7.69)	<0.001
70 years and more	7.22 (6.14–8.49)	<0.001	5.97 (5.10–7.00)	<0.001	6.23 (5.19–7.47)	<0.001	5.26 (4.40–6.29)	<0.001
**Low physical activity (<600 METs)**								
No	Reference		Reference		Reference		Reference	
Yes	1.10 (1.01–1.19)	0.025	1.09 (1.01–1.19)	0.035	1.10 (1.01–1.19)	0.027	1.09 (1.01–1.19)	0.036
**Current daily cigarette smoking**								
No	Reference		Reference		Reference		Reference	
Yes	1.01 (0.87–1.17)	0.898	0.92 (0.79–1.06)	0.230	0.99 (0.85–1.14)	0.849	0.89 (0.78–1.03)	0.123
**BMI**								
<25 kg/m^2^	Reference				Reference			
25–29.9 kg/m^2^	2.07 (1.87–2.28)	<0.001			2.07 (1.88–2.29)	<0.001		
≥30 kg/m^2^	3.73 (3.33–4.17)	<0.001			3.70 (3.31–4.15)	<0.001		
**Abdominal obesity (WC ≥102 cm (men); ≥88 cm (women))**								
No			Reference				Reference	
Yes			2.44 (2.23–2.66)	<0.001			2.41 (2.21–2.64)	<0.001
**Educated years**								
Illiterate					Reference		Reference	
1–6 years					0.85 (0.75–0.96)	0.008	0.87 (0.77–0.99)	0.028
7–12 years					0.81 (0.71–0.93)	0.003	0.84 (0.74–0.97)	0.015
13 years and more					0.68 (0.58–0.80)	<0.001	0.69 (0.59–0.81)	<0.001

Model 1: Statistical predictors were resident area, low physical activity, current daily cigarette smoking, BMI categories or abdominal obesity adjusted for age and sex.

Model 2: Statistical predictors were resident area, low physical activity, current daily cigarette smoking, educated years, BMI categories or abdominal obesity adjusted for age and sex.

## Discussion

This nation-wide study found an alarming prevalence of metabolic unhealthiness and rising BMI rates especially among females, the urban population, and people aged 65–69 years. Based on our results, blood markers such as glucose, TGs and HDL-C as well as blood pressure, WC, and physical activity are more accurate measures of health than BMI.

Based on our results, the prevalence of metabolic unhealthiness among Iranians with normal weight and overweight phenotypes were higher than the US (11.5% vs. 9.3%, and 22.2% vs. 17.2%, respectively). The prevalence rate of metabolic unhealthiness among obese Iranians was lower than the US (18.3% vs. 24.8%) [9]. It is important to acknowledge that the prevalence of metabolic healthiness/unhealthiness varies from study to study based on used definition. Several sets of criteria have been used to define these phenotypes, some of which are considered as indicators of metabolic disorders such as, T2DM, dyslipidemia, and hypertension with/without insulin resistance, whereas others are considered as inflammatory markers [8, 20, 21]. The use of different definition makes the comparison of the prevalence rates of these phenotypes as well as the assessment of their long-term health effects between studies difficult. In addition, the cutoff values for each parameter also vary in the studied populations due to differences in their risk distributions [8, 22, 23]. The reasons behind the differences found between our results and that of the US are the differences between the age and race of the participants as well as the used criteria. Our participants were older (mean age: 47.6 years) than the US population (45.0 years in Wildman et al.’s study) [23]. Similar to our previous study on MetS prevalence in Iran [13], the rate of metabolic unhealthiness increased with age both in Iran and the US [24], confirming that the declining prevalence of MHO with age is independent of the criteria used to define MHO. Our samples were limited to subjects with the Caucasian ethnicity, whereas the NHANES study included different races, non-Hispanic whites and blacks as well as Hispanic and Mexican American adults [8]. Moreover, unlike the US study, insulin and high-sensitive-C reactive protein (hs-CRP) levels were not measured in our study.

We found a significant negative association between BMI categories and the risk of being metabolically unhealthy in both normal weight and overweight/obese phenotypes and also in subjects with low physical activity. For instance, physical activity is expected to have positive effects on cardiometabolic risk factors even among overweight and obese subjects [8, 17, 25, 26]. The prevalence of metabolic unhealthiness, regardless of BMI phenotype, was lower in our participants with higher levels of education. The association between the prevalence of obesity and other MetS criteria and educational level is well known [13, 27–29]. Compared with BMI-derived obesity, a higher prevalence of metabolic unhealthiness was found when having abdominal obesity was considered rather than BMI. This result indicates that visceral fat might be more relevant to metabolic abnormalities than BMI [30, 31]. Although some believe that WC should not be used to define MHO as some obese subjects also have high WC [31], our study showed a negative association between having larger WC and MHO (based on BMI). The correlation noted between metabolic abnormalities and years of education in our study being independent of BMI and WC could point out the cultural differences between the US and our population as another reason behind the variety in the reported prevalence rates.

In agreement with the Finnish cohorts [32], the highest prevalence of MetS was seen among our obese subjects. This finding confirms the increase in MetS prevalence rate with surging BMI. However, in some studies conducted in the Italian and Dutch populations, the prevalence of MetS was not reported to be higher among obese individuals [32, 33]. The most frequent MetS component in obese Finnish subjects was elevated blood pressure [32]. In contrast, lipid abnormality was the most frequent MetS component among our obese individuals. As a result, dyslipidemia could be concluded as the main contributing factor to unhealthy obesity and MetS among the Iranian population. Furthermore, previous reports on the prevalence of dyslipidemia in Iran (43.9%) [34] could confirm the ethnical predisposition of Iranians to insulin resistance along with heavy consumption of high calorie unhealthy foods and the sedentary lifestyle in this population [35, 36].

After 5.5 to 10.3 years of follow-up, it has been established that MHO is not a static condition and can transform into metabolic unhealthiness over time [37, 38]. It is, therefore, of great importance to define the variables that could predict the transition from metabolically healthy to unhealthy in each population. In our study, the frequency of MetS components increased with BMI regardless of sex. In addition, low physical activity and central obesity enhanced the transition from being metabolically healthy to unhealthy phenotype. In the Spanish population, any increase in BMI, WC or waist-to-hip-ratio was reported to help accelerate this transition, whereas following a healthy diet, high levels of physical activity, not smoking or cessation of smoking helped stop the shift [39]. Conversely, factors such as the female gender, high insulin levels, low levels of HDL-C, and greater visceral fat accumulation were the factors accelerating this change in the Japanese Americans [40].

Our study had several strengths and limitations. The main strength was its population-based sampling nature that was representative of the Iranian population and the precise characterization of the participants. Moreover, the accurate estimation of BMI-derived obesity through direct measurement by trained research staff along with the estimation of metabolically healthy/unhealthy prevalence rates across different provinces of Iran were among other strengths. The cross-sectional design of the study, on the other hand, limited the establishment of causal associations. Given the global rise of obesity, the comparison of metabolically healthy/unhealthy prevalence rates based on our data (collected in 2016) with that of the NHANES 1999–2004 data could show bias because of the differences mainly in the sampling time.

### Conclusion

By focusing on BMI alone, the identification of overweight and obese individuals who are metabolically healthy as well as metabolically unhealthy subjects with normal weight is not possible. In other words, the “one size fits all” management approach could prove ineffective and should not be recommended for every overweight and obese individual. In addition, due to its heterogeneity, an expert consensus is urgently required to standardize the definition of MHO. This could be performed through considering a set of criteria including insulin resistance, T2DM markers, hypertension, dyslipidemia, inflammatory biomarkers such as hs-CRP as well as WC. Moreover, it should be remembered that MHO is a dynamic concept that can alternate to a metabolically unhealthy condition over time. So, more focus needs to be laid on variables that could help prevent this transition.

Nevertheless, given the high mortality rate attributable to excess BMI and due to ischemic heart diseases, stroke and T2DM in Iran [41] and lack of applicable obesity prevention programs, appropriate intervention programs are required. In this regard, further studies are needed to identify the factors contributing to the differences between provinces, especially among neighboring provinces. Given the high prevalence of dyslipidemia among obese subjects, raising awareness about lipid abnormalities, healthy lifestyles, lipid-lowering medications as well as expanding lipid clinics could be an important intervention.

## Supporting information

S1 Fig National prevalence of metabolic phenotypes for each BMI category by metabolic syndrome components classified by sex and area of residence. (TIFF)

## List of abbreviations

MHO: Metabolically healthy obesity
BMI: Body mass index
MetS: Metabolic syndrome
NCEP ATP III: National Cholesterol Education Program Adult Treatment Panel III 2004
NCDs: Non-communicable diseases
T2DM: Type 2 diabetes mellitus
CVD: Cardiovascular diseases
MUHNW: Metabolically unhealthy normal weight
NHANES: National Health and Nutrition Examination Survey
NIMAD: National Institute for Medical Research Development
WC: Waist circumference
WHR: Waist-to-hip-ratio
SBP: Systolic blood pressure
DBP: Diastolic blood pressure
GPAQ: Global Physical Activity Questionnaire
MET: Metabolic Equivalent of Task
FBS: Fasting blood sugar
TChol: Total cholesterol
HDL-C: High density lipoprotein cholesterol
LDL-C: Low density lipoprotein cholesterol
TGs: Triglycerides
NCDRC: Non-Communicable Diseases Research Center
MHUW: Metabolically healthy underweight phenotype
MUHUW: Metabolically unhealthy underweight phenotype
MHNW: Metabolically healthy normal weight phenotype
MUHNW: Metabolically unhealthy normal weight phenotype
MHOW: Metabolically healthy overweight phenotype
MUHOW: Metabolically unhealthy overweight phenotype
MUHO: Metabolically unhealthy obese phenotype
95% CI: 95% confidence interval
OR: Odds Ratio
hs-CRP: High-sensitive-C reactive protein
