# Supplementary figures and images for: Association between being metabolically healthy/unhealthy and metabolic syndrome in Iranian adults
Source: PLoS One. 2022 Jan 6;17(1):e0262246. doi: 10.1371/journal.pone.0262246 (PMC8735615; doi:10.1371/journal.pone.0262246)

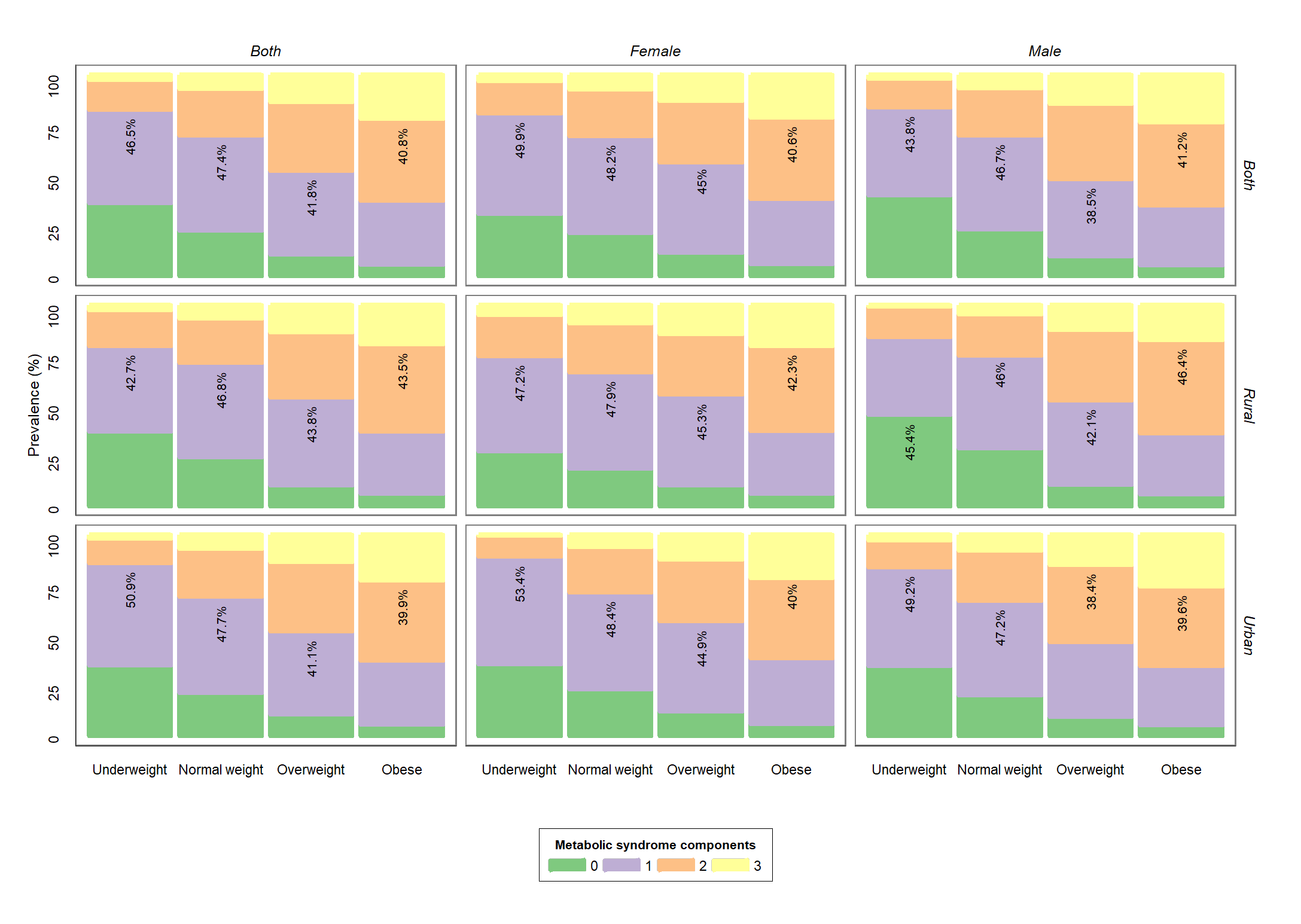

Supplement: S1 Fig — (TIFF) [file pone.0262246.s001.tiff]
